# *In vitro* function of random donor platelets stored for 7 days in composol platelet additive solution

**DOI:** 10.4103/0973-6247.75969

**Published:** 2011-01

**Authors:** Ashish Gupta, Tulika Chandra, Ashutosh Kumar

**Affiliations:** 1 *Department of Transfusion Medicine and Blood Bank, Chhatrapati Shahuji Maharaj Medical University, Lucknow, Uttar Pradesh, India*; 2 *Department of Pathology, Chhatrapati Shahuji Maharaj Medical University, Lucknow, Uttar Pradesh, India*

**Keywords:** Additive solution, *in vitro*, random donor platelets, storage

## Abstract

**Background and Aim:**

Platelets are routinely isolated from whole blood and stored in plasma for 5 days. The present study was done to assess the *in vitro* function of random donor platelets stored for 7 days in composol platelet additive solution at 22°C.

**Materials and Methods:**

The study sample included 30 blood donors of both sex in State Blood Bank, CSM Medical University, Lucknow. Random donor platelets were prepared by platelet rich plasma method. Whole blood (350 ml) was collected in anticoagulant Citrate Phosphate Dextrose Adenine triple blood bags. Random donor platelets were stored for 7 days at 22°C in platelet incubators and agitators, with and without additive solution.

**Results:**

Platelet swirling was present in all the units at 22°C on day 7, with no evidence of bacterial contamination. Comparison of the mean values of platelet count, platelet factor 3, lactate dehydrogenase, pH, glucose and platelet aggregation showed no significant difference in additive solution, whereas platelet factor 3, glucose and platelet aggregation showed significant difference (*P* < 0.001) on day 7 without additive solution at 22°C.

**Conclusion:**

Our study infers that platelet viability and aggregation were best maintained within normal levels on day 7of storage in platelet additive solution at 22°C. Thus, we may conclude that *in vitro* storage of random donor platelets with an extended shelf life of 7 days using platelet additive solution may be advocated to improve the inventory of platelets.

## Introduction

Platelets are small, anucleated cytoplasmic fragments that play an essential role in blood clotting and wound healing.[1] Platelets are routinely isolated from whole blood and stored in plasma for 5 days. Furthermore, the use of platelet additive solution could help to improve storage conditions and thus increase the shelf life of the platelets while maintaining the viability and hemostatic function.[2,3] The present study was done to assess the *in vitro* function of random donor platelets stored for 7 days in composol platelet additive solution at 22°C.

## Materials and Methods

The study sample included 30 blood donors of both sex in State Blood Bank, Department of Transfusion Medicine, Chhatrapati Shahuji Maharaj Medical University, Lucknow. Complete medical history of donors was taken to exclude any infection and disease in the collected samples.

### Subjects studied

The blood donors were selected after taking a detailed history and a complete examination regarding their eligibility criteria for blood donation. Donor’s name, age, sex, occupation, caste, complete postal address and contact number were recorded. Donors were deferred or accepted according to their medical history regarding chronic or acute diseases. Findings were further confirmed by physical examination of the donor. Blood was taken from a donor only after they were found to fulfill all the eligibility criteria of a healthy donor. Written consent was also taken from them prior to donation, regarding their acceptability for the tests to be carried out for the transfusion transmitted diseases as well for the platelet function studies.

### Random donor platelets preparation

Random donor platelets were prepared by platelet rich plasma (PRP) method.[4] Whole blood (350 ml) was collected in anticoagulant Citrate Phosphate Dextrose Adenine (CPDA) triple blood bags (HL Hemopack, Hindustan Latex Ltd., Kerala, India). After a resting time of 30 minutes, whole blood was centrifuged in a Cryofuge 6000i (Heraeus-Kendro, Hanau, Germany) at 1750 ×g for 8 minutes at 22°C to obtain PRP. The obtained PRP was again centrifuged at 3850 ×g for 8 minutes under the same experimental conditions. After the final centrifugation, the supernatant platelet poor plasma (PPP) was separated, and the residual pellet with the platelets was resuspended in a mean volume of 50 ± 0.9 ml of plasma and the remaining 150 ml of plasma was removed. Random donor platelets were divided into two parts by a sterile tubing welder (Terumo TSCD, SC-201 AH, Leuven, Belgium). Four milliliters of additive solution was added to 30 units each of the random donor platelets. The bags were placed in a platelet incubator with agitator (Remi Instruments Ltd., Mumbai, India). Random donor platelets were evaluated on day 0, day 5 and day 7 at 22°C of storage with and without additive solution.

### Preparation of random donor platelets using platelet additive solution (composol)

All the random donor platelets were stored in additive solution which had been standardized using the following constituents: sodium chloride (5.26 g) (Merck Pvt. Ltd., Mumbai, India), sodium gluconate (5.02 g) (Rolex Chemicals Industries, Mumbai, India), sodium acetate anhydrous (2.22 g) (Ranbaxy Fine Chemicals Ltd., New Delhi, India), potassium chloride (0.373 g) (Ranbaxy Fine Chemicals Ltd., New Delhi, India), magnesium chloride hexahydrate (0.305 g) (Merck Pvt. Ltd., Mumbai, India), and sodium citrate (3.213 g) (Sisco Research Pvt. Ltd., Mumbai, India). All the constituents were dissolved in 1000 ml of distilled water and steam sterilized. The pH of this additive solution was 7.2.[5]

### Screening of blood and storage of platelet units

All the blood units were screened for Hepatitis B virus (Hepalisa, J Mitra and Co. Pvt. Ltd., New Delhi, India), Hepatitis C virus (HCV Microlisa, J Mitra and Co. Pvt. Ltd., New Delhi, India), and Human Immunodeficiency Virus 1 and 2 (Microlisa-HIV, J Mitra and Co. Pvt. Ltd., New Delhi, India). The method used was enzyme-linked immunosorbent assay (Elisa plate washer version 3 and Elisa plate reader version no. 1.300, Robonik Pvt. Ltd., Navi Mumbai, India). Syphilis was tested by Rapid Plasma Reagin (RPR) method (Span Diagnostic Ltd., Surat, India). Random donor platelets were stored at 22°C in platelet incubators and agitators. Thirty units each of random donor platelets were stored at 22°C with and without additive solution.

### Assessment of platelet count and functions

Standard protocols were followed to assess platelets on day 0, day 5 and day 7 of storage. Samples were withdrawn under sterile conditions in biosafety cabinet grade 2. Platelet count was done using automated cell counter (MS4, Blood cell counter, Anand Group, HD Consortium, Bangalore, India). Platelet functions were assessed by platelet factor 3 (PF-3) with kaolin and CaCl_2._[6] Exactly 100 μl of platelet rich test plasma was added to 100 μl of platelet poor normal plasma in a test tube held at 37°C in a water bath. 200 μl of kaolin was added and the stopwatch was started. The mixture was incubated for 20 minutes with occasional shaking and then 200 μl of CaCl_2_ was added and clotting time was recorded with a second stopwatch. The procedure was repeated with a mixture of 100 μl of platelet poor test plasma and 100 μl of platelet rich normal plasma. Lactate dehydrogenase (LDH) determination was conducted on random donor platelet samples as follows. Random donor platelets (1 ml) were centrifuged at 3000 ×g for 5 minutes. The supernatant was used to quantify the LDH by semi-automated Microlab 300 (Merck Specialties Pvt. Ltd., Goa, India). Glucose determination was done by centrifuging 2 ml of random donor platelets in fluoride oxalate vial at 3000 ×g for 5 minutes. The supernatant was used to quantify the glucose by Erbachem 5 Plus analyzer (Erba diagnostic Mannhein Gmbh, Mannhein, Germany). The pH of all samples was assessed immediately after sampling at a temperature of 24°C by Compla pH meter (Composite Lab Line Pvt. Ltd., Lucknow, India). Platelet aggregation was determined by optical method[7] using a flat-bottom aggregometer (Chornolog Corporation, Havertown, PA, USA). The functional study of random donor platelets was performed by platelet aggregation, using 5 μM adenosine di phosphate (ADP) as agonist (Amresco, Solon Ind. Pkwy. Solon, OH, USA) at different storage periods. Aerobic culture was performed on all the samples on day 0, day 5and day 7 using manual method of culture.[8] The readings at day 5 and day 7 were analyzed taking day 0 as control.

### Statistical analysis

Data were reported as means ± standard deviation (SD). The data were compared using paired t-test. The confidence limit was kept at 95%, hence a *P* value <0.05 was considered to be statistically significant.

## Results

None of the samples showed bacterial contamination on day 7 at 22°C ± 0.5 with and without additive solution. On comparing the mean values of platelet count in both groups, no significant difference was observed on day 7 of storage period. On assessment of platelet factor 3, no significant difference was seen on day 7 with additive solution. In contrast, a significant difference was observed on day 7 (*P* < 0.001) without additive solution. The mean values of LDH and pH showed no significant difference on day 7 in both the groups. A significant difference was observed in the levels of glucose on day 7 (*P* < 0.001) without additive solution. In platelet aggregation also, a significant decrease was seen without additive solution (*P* < 0.001) on day 7 at 22±C [Table 1].

**Table 1:** Comparison of the parameters of random donor platelets stored for 7 days at 22°C with and without additive solution

Parameter	Platelet with additive solution			Platelet without additive solution		
	0 day	5 day	7 day	0 day	5 day	7 day
Platelet count (m/mm^3^)	245 ± 34	242 ± 32	239 ± 34	245 ± 30	240 ± 30	236 ± 34
Bacterial contamination	Sterile	Sterile	Sterile	Sterile	Sterile	Sterile
PF-3 (seconds)	2 ± 1	2 ± 1	2 ± 1	2 ± 1	2 ± 1	3 ± 1*
LDH (U/l)	132 ± 24	134 ± 22	139 ± 24	130 ± 23	134 ± 21	137 ± 22
pH	7.15 ± 0.03	7.13 ± 0.04	7.12 ± 0.05	7.16 ± 0.05	7.15 ± 0.02	7.12 ± 0.04
Glucose (mmol/l)	10.9 ± 2	10.0 ± 4	9.2 ± 4	13.4 ± 2	12.1 ± 2	7.6 ± 4*
Platelet aggregation % (5 μM ADP)	72 ± 4	63 ± 4	56±3	74 ± 4	60 ± 4	48 ± 3*

*n* = 30 units of random donor platelets; values are mean ± SD

* *P* < 0.001 compared to 5 day samples

## Discussion

Optimized synthetic storage media might help attenuate the platelet storage lesion, thereby facilitating extended storage. Composol contains acetate, which serves as a second metabolic fuel. Acetate has the added benefit of acting as a buffer. Magnesium and potassium are present in composol. These electrolytes inhibit platelet activation and aggregation, although it is not clear as to how they work.[9] Potassium plays an important role in maintaining the platelet membrane potential[10] and when absent, potassium will leak rapidly from the platelet and needs to be recovered by energy-requiring potassium pumps. Furthermore, it has been shown that presence of external magnesium activates various potassium pumps.[11] Also, there is evidence that magnesium decreases the platelet activation[12] and influences the calcium influx into the platelets, thereby having an effect on the intracellular concentration of potassium.[13]

Platelets can be prepared using random donor platelets, apheresis and by pooling of platelet units.[4] Random donor platelets were used in the present study. Platelet swirling was present in all the units at a temperature of 22±C with and without additive solution on day 7. No evidence of bacterial contamination was found on day 7 in both the groups. In the present study, platelet count was maintained on day 7 at 22±C in both the groups.

Shortening of the clotting time of intact PRP by incubation with celite or kaolin is believed to result from activation of the Hageman and plasma thromboplastin antecedent (PTA) coagulation factors and from release of platelet factor 3. It is possible to control the test system so that it is sensitive only to the increase in platelet coagulant activity.[14] Platelet factor 3 may also be released by antiplatelet antibodies. However, the kaolin test has the important advantage of extreme simplicity both in apparatus and in performance. Hence, it is valuable to do a more rigorous standardization of the kaolin test, with an attempt to narrow the observed normal range and thus improve clinical discrimination. The two mixtures (platelet rich test plasma and platelet poor normal plasma) differ only in the platelets they contain and clotting time should not differ by more than 2 or 3 seconds. A prolongation of the clotting time of the mixture containing the test platelet compared to that containing the normal platelets is an evidence of reduced platelet factor 3 availability. It is desirable to measure the clotting times of mixture of platelet rich and platelet poor samples of the test plasma and normal plasma, respectively. In the present study, Platelet factor 3 variation was within 3 seconds at 22±C even on day 7 in both the groups. The fact that platelet fragments can retain their procoagulant activity lends credence to the commonly held theory that platelet factor 3 generation is dependent on configurational changes of the platelet membrane.[15]

In the present study, it was observed that the LDH level slightly increased on day 7 in random donor platelets with and without additive solution and also that the level of LDH was maintained on day 7at 22±C. The American Association of Blood Banks (AABB)[16] recommended that platelets with pH <6.2 should not be used for transfusion, and in Europe, the same recommendation applies to platelets with pH >7.4.[17] As per the Drug and Cosmetics Act of India,[18] minimum pH should not be <6.0 at any given day of storage. If the pH falls below 6.0 or rises above 7.4, a disk to sphere transformation of the platelets takes place, resulting in marked loss of recovery *in vivo* upon transfusion.[19] In the present study, we observed that the pH value decreased and maintained within acceptable range on day 7 at 22±C in the storage period of both the groups. van der Meer *et al*.[20] made a comparison between two platelet additive solutions: one containing citrate and acetate (PAS-II), and the other also supplemented with additional salts such as magnesium, and with gluconate (composol-PS). The platelet concentrates were prepared by pooling five buffy coats and the additive solution, and prestorage filtration was utilized to remove leukocytes to well below 1 × 10^6^. Storage of platelet concentrates up to 9 days after blood collection revealed that platelet concentrates in composol-PS maintained an almost constant pH at an average 6.93 from day 2 through day 7, and at 6.90 at day 9. This was in contrast to PAS-II which showed a gradually decreasing pH from an average 6.97 at day 1 to 6.86 at day 9. In all units stored in both the solutions, the swirling effect was present during 9 days of storage. They concluded that both the additive solutions allow storage of platelets, derived from pooled buffy coats, for up to 9 days after collection of the whole blood, with maintenance of good quality *in vitro*. Composol-PS has a slightly better buffering capacity, reflected as a more constant pH throughout the storage period.

In the present study, the glucose level slightly decreased in random donor platelets with and without additive solution on day 7at 22°C. One of the most common methods of measuring platelet aggregation is called optical platelet aggregation. This technique, which is a high-complexity laboratory test, involves adding an aggregating agent (e.g., ADP, epinephrine, thrombin, arachidonic acid) to PRP, a turbid platelet-rich suspension derived from whole blood. The effect of the aggregating agent on the suspension’s light transmittance is then measured to assess platelet aggregation.[7] In the present study, we observed that platelet aggregation was decreased in random donor platelets with and without additive solution on day 7 at 22±C. Kiraly *et al*. (2006)[21] studied the functional viability parameters of single donor platelets for 5 days at room temperature with agitation. They also assessed a number of *in vitro* parameters (pH, morphology, platelet volume distribution, osmotic recovery, aggregation and platelet associated IgG) as a function of storage time of platelets. During the first 24 hours of storage, minimal changes were observed in the test parameters with the exception of ADP-induced aggregation [(75% decrease (10 μM), 84% decrease (5 μM)]. Significant difference was observed between day-0 and day-5 old single donor platelets in all the parameters.

Overall, the results showed that all the parameters were maintained in random donor platelets stored for 7 days in platelet additive solution at 22±C. van der Meer *et al*.[22] compared the *in vitro* storage of characteristics of pooled buffy coat platelets stored for up to 12 days in 100% plasma, or in mixtures of plasma with PAS-II, PAS-III, PAS-III, PAS-IIIM and composol. They observed that several *in vitro* markers of platelet quality (pH ≥ 6.8, glucose consumption, lactate production) were reasonably well preserved for 9-12 days in platelets stored either in 100% plasma or in PAS-IIIM (30% plasma) or composol (35% plasma).

## Conclusion

Our study infers that platelet viability and aggregation were best maintained within normal levels on day 7 of storage in platelet additive solution at 22±C. Thus, we may conclude that *in vitro* quality of random donor platelets is maintained with an extended shelf life of 7 days using platelet additive solution. This forms the basis for increasing the shelf life of platelets to 7 days from the current guidelines of 5 day storage.
